# Trodusquemine (MSI-1436) Restores Metabolic Flexibility and Mitochondrial Dynamics in Insulin-Resistant Equine Hepatic Progenitor Cells (HPCs)

**DOI:** 10.3390/cells13020152

**Published:** 2024-01-14

**Authors:** Badr Qasem, Agnieszka Dąbrowska, Jarosław Króliczewski, Jacek Łyczko, Krzysztof Marycz

**Affiliations:** 1 Department of Experimental Biology, Faculty of Biology and Animal Science, Wrocław University of Environmental and Life Sciences, Norwida 27B, 50-375 Wrocław, Poland; badr.qasem@upwr.edu.pl (B.Q.); agnieszka.dabrowska@upwr.edu.pl (A.D.); jaroslaw.kroliczewski@upwr.edu.pl (J.K.); 2 Department of Food Chemistry and Biocatalysis, Wrocław University of Environmental and Life Sciences, 50-375 Wrocław, Poland; jacek.lyczko@upwr.edu.pl; 3 Department of Medicine and Epidemiology, School of Veterinary Medicine, University of California, Davis, CA 95516, USA

**Keywords:** MSI-1436, Eq_HPCs, glucose uptake, palmitate, mitochondrial dynamics/mitophagy

## Abstract

Equine metabolic syndrome (EMS) is a significant global health concern in veterinary medicine. There is increasing interest in utilizing molecular agents to modulate hepatocyte function for potential clinical applications. Recent studies have shown promising results in inhibiting protein tyrosine phosphatase (PTP1B) to maintain cell function in various models. In this study, we investigated the effects of the inhibitor Trodusquemine (MSI-1436) on equine hepatic progenitor cells (HPCs) under lipotoxic conditions. We examined proliferative activity, glucose uptake, and mitochondrial morphogenesis. Our study found that MSI-1436 promotes HPC entry into the cell cycle and protects them from palmitate-induced apoptosis by regulating mitochondrial dynamics and biogenesis. MSI-1436 also increases glucose uptake and protects HPCs from palmitate-induced stress by reorganizing the cells’ morphological architecture. Furthermore, our findings suggest that MSI-1436 enhances 2-NBDG uptake by increasing the expression of SIRT1, which is associated with liver insulin sensitivity. It also promotes mitochondrial dynamics by modulating mitochondria quantity and morphotype as well as increasing the expression of PINK1, MFN1, and MFN2. Our study provides evidence that MSI-1436 has a positive impact on equine hepatic progenitor cells, indicating its potential therapeutic value in treating EMS and insulin dysregulation.

## 1. Introduction

The liver is an essential and multifunctional organ that plays a pivotal role in maintaining metabolic homeostasis in humans and animals [1]. It consists of hepatocytes and biliary epithelial cells, which differentiate from common progenitor cells called hepatoblasts during embryonic development [2]. Hepatocytes are the primary cell type in the liver, constituting up to 80% of all hepatic cells. These cells perform diverse functions, including the detoxification of metabolites, regulation of glucose and lipid metabolism, synthesis of serum proteins, and secretion of bile [3,4,5,6]. Non-alcoholic fatty liver disease (NAFLD), a prevalent chronic liver disorder, is characterized by abnormal accumulation of triglycerides and other lipids in the liver. Liver disorders are strongly associated with insulin resistance, type 2 diabetes, obesity, and metabolic syndrome [3,7].

It has been reported that severe or prolonged liver disorders hinder the effective regeneration of hepatocytes from residual cells [8]. In such cases, hepatic progenitor cells (HPCs) are stimulated and undergo clonogenic proliferation and differentiation into various lineages, including hepatocytes and bile ductal epithelia [9]. Additionally, these HPCs migrate from the portal vein area to the liver parenchyma and differentiate into fully functional hepatocytes and bile duct cells, facilitating the restoration of the damaged liver [10]. The evidence presented underscores the potential of HPCs as a valuable model for investigating liver diseases and regeneration, as well as for developing novel therapeutic approaches. Equine metabolic syndrome (EMS) is a complex disorder characterized by insulin resistance, hyperinsulinemia, hyperleptinemia, increased adiposity, and inflammation. It is strongly associated with the development of laminitis, a life-threatening condition in horses [11,12,13]. Despite extensive research efforts, managing EMS continues to pose a significant challenge in veterinary medicine.

Recent evidence indicates that excessive consumption of high-carbohydrate (NSC) forage promotes hepatic de novo lipogenesis and leads to lipotoxicity. Dysregulation of glucose metabolism can also contribute to the development of insulin resistance, which is significant factor in the pathogenesis of equine metabolic syndrome (EMS) [14,15,16,17,18]. The binding of insulin to the insulin receptor (INSR), a tyrosine kinase receptor, initiates a series of phosphorylation events that activate intracellular signaling pathways responsible for transporting glucose into the liver through the GLUT2 transporter [19,20]. GLUT2 helps maintain a balance between intracellular and extracellular glucose levels through facilitated diffusion [21]. Growing evidence suggests that SIRT1 plays a critical role in regulating hepatic lipid metabolism [22]. Additionally, SIRT1 activation can prevent liver steatosis by inducing the expression of fibroblast growth factor 21 (FGF21), a hormone produced by hepatocytes that restores glucose and lipid homeostasis in obesity-induced diabetes [23]. Importantly, both the SIRT1 and mTOR pathways converge on common downstream targets that are crucial for longevity regulation in various organisms, including mice. Recent evidence suggests that SIRT1 can negatively modulate mTOR signaling, potentially by inhibiting tuberous sclerosis complex 1/2 (TSC 1/2) [24,25].

Moreover, the expression of PTP1B has been associated with the homeostasis of liver tissue and serves as a physiological factor that distinguishes metabolic disorders [26]. PTP1B is known to exert a negative regulatory effect on leptin and insulin signaling, which has implications for insulin resistance and metabolic disorders [27]. Our previous study has also indicated that PTP1B plays a role in the activation of hepatic stellate cells (HSCs), which contribute to the excessive accumulation of extracellular matrix in liver fibrosis [27]. Trodusquemine (MSI-1436), a natural spermine–cholesterol adduct, acts as an inhibitor of protein tyrosine phosphatase 1B (PTP1B). It specifically and reversibly inhibits PTP1B by targeting its long form (1–405), which includes an extended C-terminal segment [28]. Previous studies conducted by our team have demonstrated that MSI-1436 effectively mitigates tunicamycin-induced ER stress by modulating XBP1 splicing. Consequently, it enhances the survival of hepatocellular carcinoma cell lines exposed to stressed conditions induced by palmitate/oleate. This protective effect is achieved by reducing lipoapoptosis, improving mitochondrial dynamics, and attenuating oxidative and endoplasmic reticulum stress [5,26]. Additionally, MSI-1436 has been shown to enhance the differentiation of adipose-derived progenitor stem cells from EMS by modulating endoplasmic reticulum stress, apoptosis, and oxidative stress [29]. 

Furthermore, insulin resistance (IR) commonly occurs in insulin-sensitive tissues such as the liver, adipose tissue, and muscle due to various mechanisms, including endoplasmic reticulum (ER) stress, impaired mitochondrial dynamics, and autophagy dysfunction [15,16,18]. Consequently, the cell has developed various quality control processes to counteract the accumulation of mitochondrial damage [30]. For instance, excessive accumulated damage leads to a severe loss of function. Mitochondria are directed to lysosomes and degraded through a process known as mitophagy, which involves the PTEN-induced putative kinase 1 (PINK1) and PARKIN pathway [31,32]. PTEN-induced putative kinase 1 (PINK1) and the E3 ubiquitin ligase Parkin play a pivotal role in regulating mitophagy [33]. Specifically, PINK1 is typically located in the outer mitochondrial membrane, but its absence in healthy mitochondria can be attributed to its cleavage by the intramembrane-cleaving protease PARL upon localization to the mitochondrial matrix. This process releases a truncated form of PINK1 into the cytoplasm where it undergoes further degradation by the ubiquitin proteasome system to maintain basal levels [34]. Additionally, PINK1 is unable to be transported into the inner mitochondrial membrane and avoid cleavage by intramembrane-cleaving protease PARL. As a result, PINK1 accumulates in the outer mitochondrial membrane where it recruits autophagy receptors such as SQSTM1/p62, nuclear dot protein 52 (NDP52), and optineurin (OPTN) to facilitate binding to LC3 and the subsequent interaction between dysfunctional mitochondria and autophagosomes [35]. Furthermore, PINK1 phosphorylates Parkin, aiding its translocation to the outer mitochondrial membrane and activating mitophagy, which contributes to the clearance of dysfunctional mitochondria [36,37]. Moreover, Parkin can induce mitophagy by promoting the ubiquitination of several proteins, including the mitochondrial fusion proteins mitofusin 1 (MFN1) and mitofusin 2 (MFN2), the mitochondrial adapter protein Miro1, translocase of the outer mitochondrial membrane 20 (TOM20), and voltage-dependent anion channel (VDAC) [33,38].

The aim of this study was to assess the effectiveness of MSI-1436 in improving glucose uptake, mitochondrial dynamics, and mitophagy in equine hepatic progenitor-like cells (Eq_HPCs) during palmitate-induced mitochondrial dysfunction, and investigate whether lipid overload in Eq_HPCs contributes to the development of equine metabolic syndrome (EMS) by diminishing their regenerative ability. 

## 2. Materials and Methods

The cell culture reagents and chemicals used in this study were purchased from Sigma Aldrich (Poznań, Poland) unless otherwise specified.

### 2.1. Cell and Culture Conditions

Equine hepatic progenitor cells (HPCs) were generated using a two-step differentiation process of equine adipose-derived stromal cells (ASCs), as previously described [7,39]. Briefly, the ASCs were subjected to a 10-day differentiation process. During the conditioning step, the cells were incubated for 2 days in DMEM-low glucose (LG) culture medium supplemented with 20 ng/mL of epidermal growth factor (EGF) and 10 ng/ mL of basic fibroblast growth factor (bFGF) (conditioning step). Subsequently, the cells were cultured for an additional 8 days in DMEM-LG supplemented with 20 ng/mL of hepatocyte growth factor (HGF), 10 ng/mL of bFGF, and 4.9 mmol/L of nicotinamide. The cultures were maintained at 37 °C, 5% CO_2_, and 95% humidity, with media change performed twice weekly.

### 2.2. In Vitro Study

The generated hepatic progenitor cells were divided into three experimental groups: control (CTRL), PA (treated with palmitic acid), and MSI (treated with PA and 1 μM of trodusquemine, MSI-1436). Before the start of the experiment, the MSI experimental group was pretreated for 24h with MSI-1436 in DMEM-LG supplemented with 1% penicillin/streptomycin mix (P/S). The control and PA groups were also incubated in standard culture conditions. Sodium palmitate at a final concentration of 2 mM prepared in FFA-free bovine serum albumin (BSA) solution was used following the previously described procedure [40]. The incubation with PA was maintained for 18 h at 37 °C. 

### 2.3. Eq_HPCs Clonogenic and Proliferation Potential

To evaluate the clonogenic potential of Eq_HPCs in each experimental group, a colony-forming unit fibroblastic (CFU-F) assay was conducted. Eq_HPCs were seeded at a density of 1 × 10^3^ cells per well and cultured for 7 days under the same conditions as described above. Afterward, the cells were stained with a pararosaniline solution, and clusters containing more than 50 cells were considered as colonies. These colonies were counted using an inverted microscope, and CFU-F was calculated using the previously described formulas [41]. Furthermore, the viability of the Eq_HPCs was assessed using a resazurin-based assay (TOX8), following the manufacturer’s protocol. Briefly, cells were seeded in 96-well culture plates at a density of 8 × 10^3^ cells/well in a total volume of 100 μL of DMEM-LG medium per well. The cells were then subjected to the various treatments mentioned above and incubated for 24 h. Subsequently, the remaining culture media were removed and 100 µL of a 10% resazurin solution was added to each well. The cells were then incubated for an additional 2 h to estimate the metabolic activity rate. Absorbance was measured using a spectrophotometer (SPECTROstar Nano, BMG LABTECH, Ortenberg, Germany) at specified wavelengths: 600 nm for resazurin and 690 nm as the reference absorbance (background). All experiments were performed with technical replicates.

### 2.4. Eq_HPCs’ Morphology Evaluation

Changes in the morphology of Eq_HPCs were analyzed under controlled experimental conditions using an inverted confocal microscope (Observer Z1 Confocal Spinning Disc V.2 Zeiss with a live imaging chamber). The evaluation of cellular morphology involved examining the localization of nuclei and assessing the development of the cytoskeleton. Initially, cells were fixed in a 4% formaldehyde solution (PFA) for 40 min, followed by permeabilization with a 0.2% Tween 20 in Hank’s Balanced Salt Solution (HBSS) for 15 min. The cytoskeleton then was stained with atto-488-labeled phalloidin (1:800) for 40 min at room temperature. Nuclei were labeled with 4′,6-diamidino-2-phenylindole (DAPI), using the ProLong™ Diamond Antifade Mountant with DAPI (Thermo Fisher Scientific, Warsaw, Poland) [42]. The obtained photomicrographs were merged and analyzed using ImageJ software (Bethesda, MD, USA). 

### 2.5. Microcapillary Flow Cytometric Analysis of Cell Viability and Apoptosis

Cell viability and apoptosis rates were assessed using theMUSE™ Annexin V & Dead Cell Kit (Merck Millipore, Darmstadt, Germany) according to the manufacturer’s instructions. Following pretreatment with MSI-1436 and exposure to PA, cells from each experimental group were harvested by trypsinization, washed, and suspended in HBSS containing 1% FBS. Subsequently, the cells were stained with the Annexin V & Dead Cell working reagent for 20 min at room temperature and analyzed using the Muse cell analyzer (Merck Millipore, Darmstadt, Germany). The total apoptotic ratio was determined by monitoring the populations of early apoptotic cells (Annexin V (+) and 7-Aminoactinomycin D (7-AAD (−))) and late apoptotic cells (Annexin V (+) and 7-Aminoactinomycin D (7-AAD (+))).

### 2.6. Cellular Glucose Uptake Analysis

Glucose uptake was examined by utilizing the fluorescent glucose analogue, 2-(N-(7-Nitrobenz-2-oxa-1,3-diazol-4-yl)Amino)-2-Deoxyglucose (2-NBDG), in accordance with the instructions provided by the manufacturer. Cells from the three distinct experimental groups (control, PA, and MSI), were treated with 100 nM insulin for a duration of 30 min, following each treatment. Subsequently, the cells were exposed to 100 µM 2-NBDG for an additional 30 min. Excess glucose analogue was removed by rinsing the cells three times with PBS, and then, they were fixed for 15 min at room temperature using 4% PFA. DAPI (Life Technologies, Warsaw, Poland) was employed to counterstain the cell nuclei in ProLong Gold Antifade and visualized using a confocal microscope (Observer Z1 Confocal Spinning Disc V.2 Zeiss with a live imaging chamber). The collected photomicrographs were further analyzed employing ImageJ software (ImageJ 1.52n, Wayne Rasband, National Institute of Health, Bethesda, MD, USA).

### 2.7. Intracellular Glucose Measured by Gas Chromatography–Mass Spectrometry (GC-MS) Analysis

The metabolites of HPCs from all treated and untreated groups were extracted using a mono-phasic mixture of chloroform/methanol/water, following the protocol outlined by Bai et al. [43] adapted for this study. Briefly, a ratio of 20:50:20 of chloroform/methanol/water (1 mL) was added to each sample in a chemical fume hood. The samples, along with the extraction reagents, were then subjected to ultrasonication at room temperature using a water bath sonicator for 100 min, followed by vortexing for 2 min. Subsequently, the samples were transferred to 1.5 mL centrifugation tubes and centrifuged at 4 °C at 18,000× *g* for 20 min. The resulting supernatants were collected for each sample and subsequently dried completely overnight. The glucose extract was washed out with two 250 µL portions of pyridine, with ultrasound assistance, and transferred into a 1.5 chromatographic vial. Then, 250 µL of N,O-bis(trimethylsilyl) trifluoroacetamide (BSTFA) was used as a silylation agent. For the derivatization process, the sample was kept for 45 min at 60 °C and shaken every 5 min. After the silylation process, the glucose concentration was determined using Shimadzu GCMS QP 2020 Plus (Shimadzu, Kyoto, Japan). A total of 2 µL of the sample was injected at 280 °C in splitless mode; helium with a linear velocity of 37.5 cm/s was used as the carrier gas. The GC temperature program was as follows: 120 °C kept for 1 min, then gradually increased to 190 °C at a rate 2 °C/min, followed by an increase to 240 °C at a rate 5 °C/min, and finally, the temperature was raised to 300 °C at a rate 10 °C. The MS operational conditions were as follows: interface temperature of 250 °C, ion source temperature of 250 °C, and scan mode of 40-1050 *m*/*z*. The identification of glucose was confirmed with a pure standard of glucose and by single ion monitoring (SIM) MS mode, which was set for 73, 147, 191, 204, and 217 *m*/*z* ions. The quantification was carried out by an external standard method. The glucose concentration obtained from each group was then normalized to the cells’ number.

### 2.8. Mitochondrial Network Staining

Mitochondria were visualized in all experimental groups using a confocal microscope (Observer Z1 Confocal Spinning Disc V.2 Zeiss with a live imaging chamber). Treated and untreated cells were incubated with the rhodamine-based MitoRed dye at a concentration of 1:1000 in complete culture medium for 30 min at 37 °C. After rinsing off excess MitoRed, cells were fixed in 4% PFA for 40 min, and coverslips were mounted on microscopic slides using the DAPI mounting medium (ProLong™ Diamond Antifade Mountant with DAPI, Thermo Fisher Scientific, Warsaw, Poland). The samples were observed using a confocal microscope at a magnification of 630× and processed with ImageJ software (ImageJ 1.52n, Wayne Rasband, National Institute of Health, Bethesda, MD, USA). 

To reconstruct the three-dimensional mitochondrial network, Leica Application Suite X (version 3.5.2.18963, Leica Microsystems CMS GmbH) was utilized. The “3D viewer” option was used to process the confocal images, and the resulting microphotographs were analyzed to determine mitochondrial morphology. This analysis was performed using MicroP software (ver. 1.1.11b, Biomedical Image Informatics Lab, Taipei City, Taiwan (R.O.C.), Institute of Biomedical Informatics, National Yang Ming Chiao Tung University), powered by MATLAB (version R2010b, MathWorks, Natick, MA, USA) [44]. The software automatically classified mitochondrial morphology based on confocal images of the mitochondrial network, categorizing them into different subtypes with quantitative analysis. To measure mitochondrial dynamics and morphology, four microphotographs were taken, capturing two cells from different cell batches at a magnification of 1000×.

### 2.9. Gene Expression Analysis

The gene expression analysis was performed following the protocol previously described by K. Kornicka-Garbowska et al. [45]. In brief, total RNA was extracted from the Eq_HPCs using TRIzol reagent, according to the manufacturer’s instructions. The purity and concentration of the extracted RNA were assessed using a nanospectrophotometer (WPA, Biowave II, Cambridge, UK). The cDNA was synthesized from the total RNA using the PrimeScript™ RT Reagent Kit with a gDNA Eraser (TaKaRa, Gdańsk, Poland), following the manufacturer’s protocol and employing a T100 Thermal Cycler (Bio-Rad, Hercules, CA, USA). Real-time reverse transcription polymerase chain reaction (RT-qPCR) was used to evaluate the gene expression levels in the Eq_HPCs. The SensiFAST SYBR Green Kit (Bioline, London, UK) was used for the reaction, which was carried out in a CFX Connect™ Real-Time PCR Detection System (Bio-Rad). The reaction mixture with total volume of 10 μL contained 5 μL of SensiFAST SYBR Master mix, 2.5 μL of targeted primer, and 2.5 μL of tested cDNA. The thermal cycle conditions were as follows: 95 °C for 2 min, followed by 40 cycles at 95 °C for 15 s, annealing for 15 s at the temperature specified for the tested primers, and elongation at 72 °C for 15 s. The expression level of the housekeeping gene glyceraldehyde 3-phosphate dehydrogenase (GAPDH) was used as a reference to determine the relative gene expression levels in the control (CTRL), PA (treated with palmitic acid), and MSI (treated with PA and 1 μM of trodusquemine, MSI-1436) samples using the 2-ΔΔCq method. Furthermore, for each RT-qPCR result, the non-treated group was used as the control for normalization. The primer sequences used are listed in Table 1.

### 2.10. Western Blot Analysis

The relative abundance of protein was investigated using Western blotting [46]. Concisely, Eq_HPCs were collected and homogenized in RIPA lysis buffer containing a cocktail of phosphatase and protease inhibitors and kept on ice. The proteins were collected by centrifuging the cell lysates for 20 min at 4 °C and 6000× *g* to remove insoluble materials and then transferred to new 1.5 mL Eppendorf tubes. The protein concentration was determined using the Pierce™ Bicinchoninic Acid (BCA) Protein Assay Kit. SDS-polyacrylamide gel electrophoresis was performed for 90 min in Tris/glycine/SDS 100 V buffer with samples diluted in 4 × Laemmli Loading Buffer and denatured at 95 °C for 5 min. The protein transfer was carried out using polyvinylidene difluoride (PVDF) membranes and a Mini Trans-Blot^®^Cell (Hong Kong, China) transfer apparatus in Tris/glycine/methanol buffer at 100 V, 250 mA, and 4 °C for 60 min. The protein membranes were blocked in a solution of 5% skimmed milk in 1X tris-buffered saline with 0.1% Tween^®^ 20 detergent (TBST) for 1 h at room temperature. Protein detection was performed by incubation overnight at 4 °C with primary antibodies (Table 2) and secondary antibodies conjugated to horseradish peroxidase (HRP), diluted 1:2500 in TBST, for 1 h at room temperature. Chemiluminescent signals were obtained using the ChemiDoc MP imaging system and quantified by using Image Lab software.

### 2.11. Statistical Analysis

The data were analyzed using GraphPad Prism 8.0.2 (GraphPad Software, San Diego, CA, USA). The results were expressed as the mean ± standard deviation (SD). To assess the normality of the data distribution (alpha = 0.05), we used the Shapiro–Wilk test and checked if the *p*-value was above 0.05. Statistical analysis of the mean differences was performed using one-way analysis of variance (ANOVA), followed by Tukey’s post-test to compare differences among the experimental groups. We considered differences to be statistically significant at * *p* < 0.05, ** *p* < 0.01, and *** *p* < 0.001, **** *p* < 0.0001.

## 3. Results

### 3.1. Evaluation of Morphology, Proliferation Rate, and Apoptosis in Eq_HPCs Treated with PA or PA/MSI-1436

**Figure 1:**
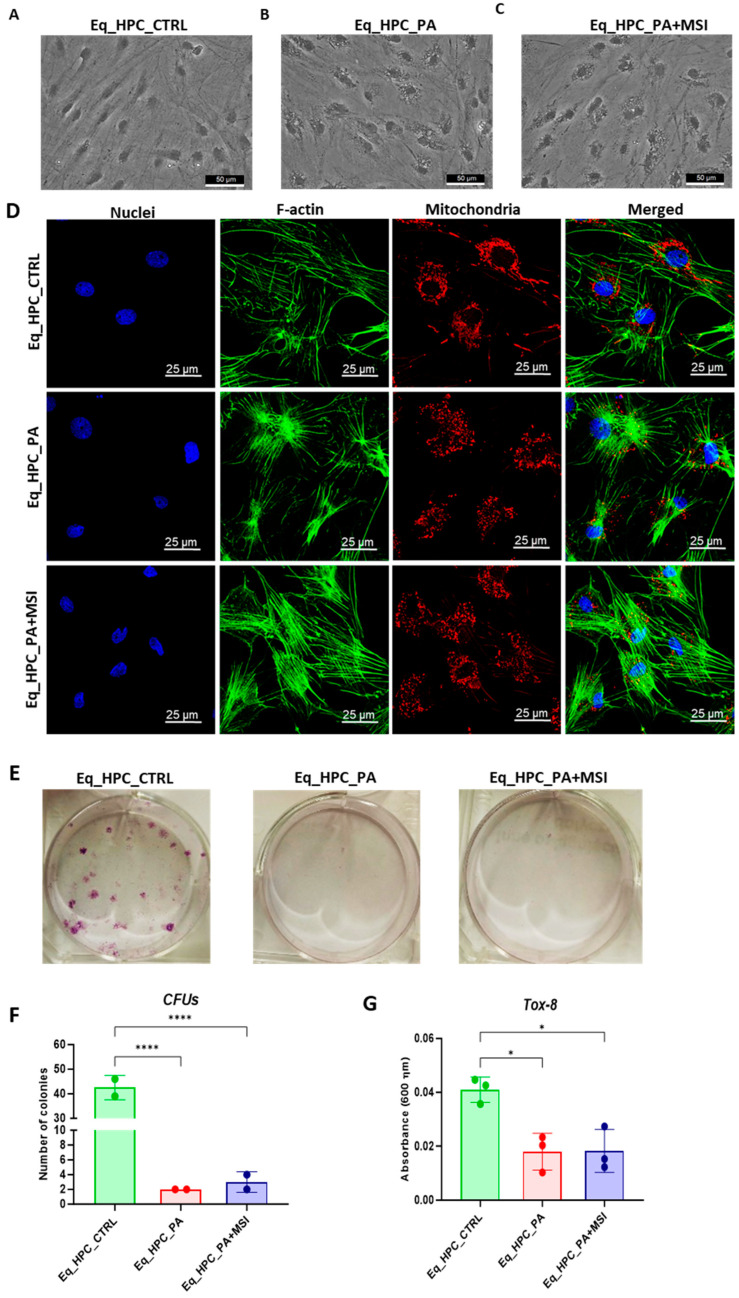

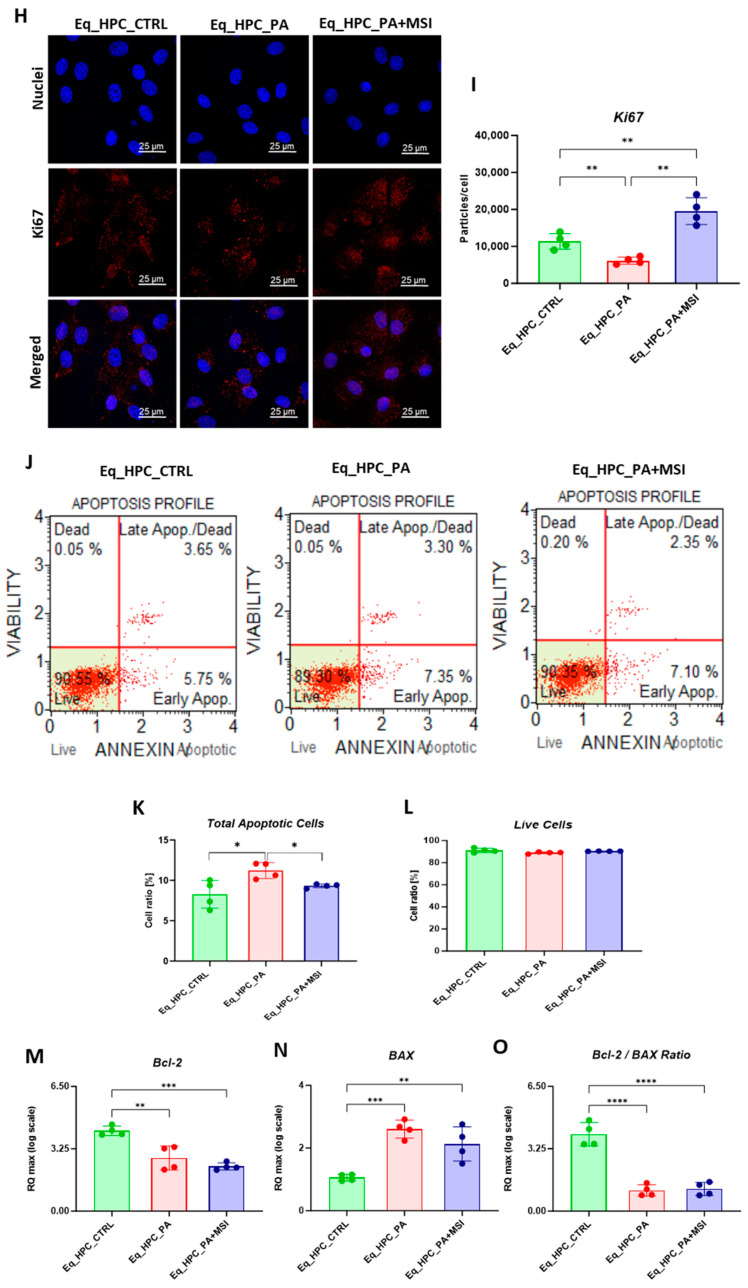
Evaluation of morphology, proliferation rate, and apoptosis. (**A**–**C**) Visualization of cells cultured in standard growth medium, cells growing from medium with the supplied PA, and the induced cells with MSI-1436 treatment. (**D**) Confocal microscope visualization of the changes in the structure and density of F-actin. (**E**–**G**) Photographs of colony-forming cells to determine CFU-F and cell viability determined by Tox-8 test. (**H**,**I**) Cell proliferative potential tested with Ki-67 staining performed with confocal microscopy. (**G**–**L**) The Muse^®^ Annexin V & Dead Cell assay results showing apoptosis in HPC_PA cells. Data were supported by the analysis of genes involved in the regulation of apoptotic pathway: Bcl-2 (**M**), BAX (**N**), and BcL2/BAX ratio (**O**). Results expressed as mean ± SD. * *p* < 0.05, ** *p* < 0.01, *** *p* < 0.001, and **** *p* < 0.0001.

The impact of PA exposure and MSI-1436 treatment on the cellular morphology of Eq_HPCs was assessed using bright-field and confocal microscopy. The results indicate that cellular stress can frequently alter the architectural organization of tissues and cells (Figure 1A–D). Control cells exhibited a typical oblong and spindle-shaped morphology. In contrast, cells cultured in standard medium, PA-treated, and MSI-treated conditions showed minimal alterations in their general shape (Figure 1A–C). However, the size of the cell bodies decreased. Additionally, numerous small granular materials were observed around the cell bodies of PA-treated and MSI-treated cells, accumulating within the cytoplasm around the nuclei. These changes suggest the presence of endoplasmic reticulum stress, cytoplasmic vacuolation, or even apoptotic fragmentation. Detailed morphological analysis of the cellular cytoskeleton and nucleus by confocal fluorescence microscopy showed that control cells exhibited an extensive and dense F-actin network with an inscrutable structure. The application of PA to Eq_HPCs resulted in a change in the shape and localization of the F-actin, with filaments concentrated around the DAPI-stained cell nuclei. However, in MSI-supplemented hepatic cells compared to the control, F-actin formed a denser and wider interconnected matrix, highlighting the morphology-preserving effects of MSI application (Figure 1D).

Furthermore, to assess the impact of PA and MSI-1436 on cell viability and survival, we performed a resazurin-based metabolic assay and the results showed that PA overload significantly decreased the number of metabolically active cells compared to the control group. However, the application of MSI-1436 to PA-stressed cells did not improve cellular viability or increase the density of metabolically active cells. The negative effect of PA on cell survival and proliferative rate was further confirmed by the colony-forming unit (CFU-F) and Tox-8 assays (Figure 1E–G).

When treating Eq_HPCs with the MSI-1436 inhibitor, we observed a slightly higher number of cellular colonies compared to untreated cells, but this difference was statistically insignificant. To further evaluate the impact of both PA and MSI-1436 on Eq_HPC growth and proliferation, we measured the levels of the nuclear antigen Ki-67 using immunofluorescence staining (Figure 1H,I). Exposure of Eq_HPCs to palmitate free fatty acid resulted in a marked decrease in the expression and abundance of the Ki-67 proliferation marker. Interestingly, cells treated with MSI-1436 showed significantly higher levels of Ki-67 protein compared to both the control group and PA-stimulated cells, suggesting the potential of MSI-1436 to induce cell cycle entry. We also tested the apoptotic tendency of all experimental groups of cells using the Muse^®^ Annexin V & Dead Cell assay (Figure 1J–L). As depicted in the Figure 1J,K, the PA-treated group showed a significantly higher number of total apoptotic cells (early and late stages; 10.6%) compared to the control cells (9.4%) and cells treated with MSI-1436 (9.5%). However, there were no significant differences in the ratio of live cells between the groups (Figure 1L). 

The mitochondrial apoptotic pathway is mediated through Bcl-2 family proteins, including both pro- and anti-apoptotic members such as Bax and Bcl-2, respectively [47]. Therefore, we examined the expression profiles of the BCL2 and BAX genes in Eq_HPCs treated with PA or MSI-1436 (Figure 1M–O). The results showed a significant reduction in BCL2 expression under both treatment conditions compared to control cells (*p* < 0.01 for PA and *p* < 0.001 for MSI) (Figure 1M). On the other hand, palmitate treatment increased the expression of BAX (Figure 1N) compared to control cells (*p* < 0.001), indicating the induction of pro-apoptotic processes. Similar trends were observed in the MSI-treated cells, where BAX expression remained significantly higher (*p* < 0.01) than that of the control cells. We also found that the BCL2/BAX ratio was highest in the control cells compared to both treatments (Figure 1O). 

### 3.2. The Effect of MSI-1436 Inhibitor on Glucose Absorption in Eq_HPCs 

**Figure 2:**
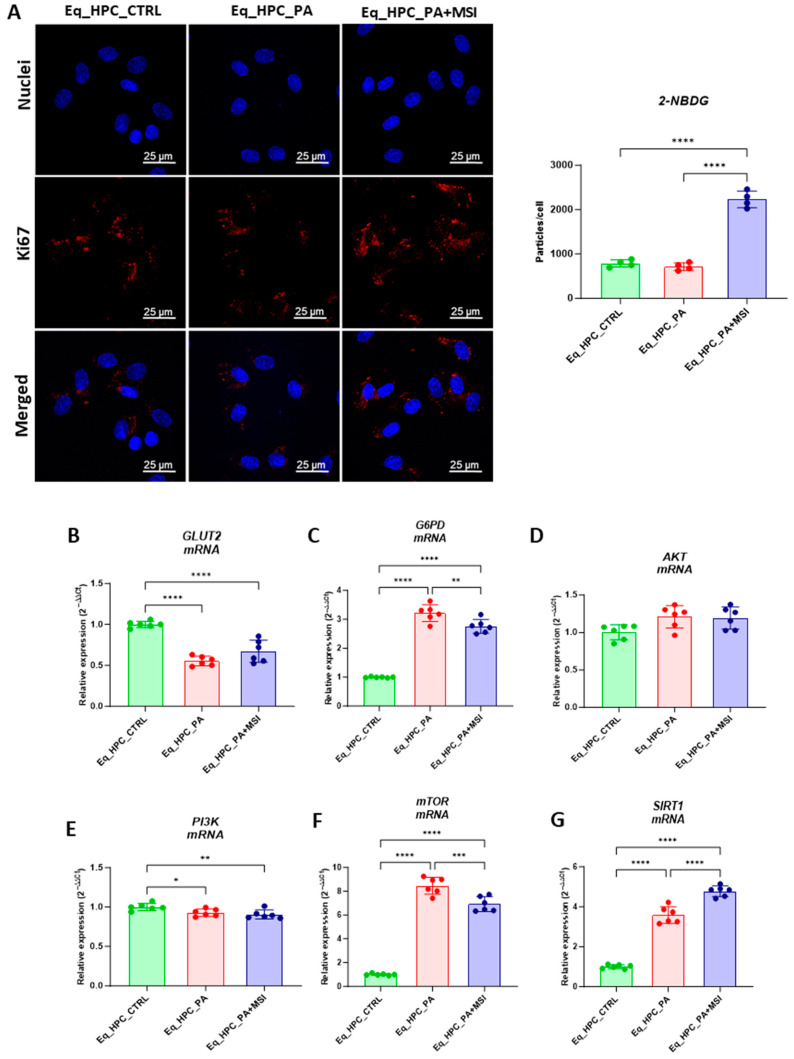

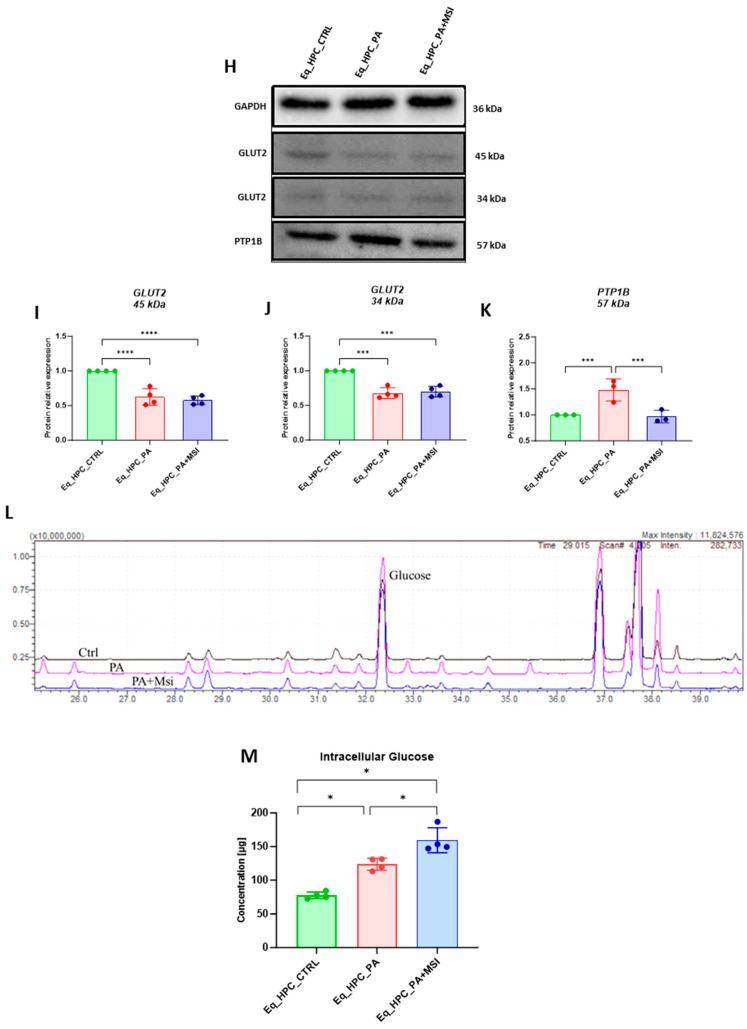
Evaluation of MSI-1436 effect on Eq_HPCs’ glucose uptake. (**A**) Representative micrographs showing the glucose uptake in Eq_HPCs measured using the 2-NBDG assay. A bar graph showing the signal counts per cell in the control and test groups. (**B**–**G**) mRNA expression of genes involved in glucose uptake. (**B**) Glut2, (**C**) AKT, (**D**) PI3K, (**E**) G6PD, (**F**) mTOR, and (**G**) SIRT1 measured with RT-qPCR. The results from experiments are normalized to GAPDH mRNA levels and expressed as a fold change over the non-treated group as the control. (**H**) Western blot analysis of GLUT2 isoforms (34 and 45 kDa) and PTPIB normalized to GAPDH (**I**–**K**). (**L**) The chromatogram of glucose-GCMS, where black chromatogram refers to control sample, pink chromatogram refers to PA samples, and blue chromatogram refers to PA+MSI samples. (**M**) The glucose concentrations (μg) obtained via GCMS analysis. The bar graph shows an analysis of relative protein expression corresponding to Western blot results. Results are expressed as mean ± SD. Significant changes (* *p* < 0.05, ** *p* < 0.01, *** *p* < 0.001, and **** *p* < 0.0001) are marked with an asterisk.

FFA accumulation and resulting lipotoxicity trigger insulin resistance and decrease hepatic glucose uptake. In this context, the effect of MSI on glucose uptake in Eq_HPCs has been investigated using a confocal microscopy approach (Figure 2A). The PA-induced stress did not contribute to a significant change in cellular glucose uptake compared to control cells. However, the inclusion of MSI in the treatment of PA-induced cells induced a statistically significant increase in glucose uptake by the cells in the study group identified by the red fluorescence glucose analog (2-NBDG) (*p* < 0.0001) (Figure 2A). These results suggest that MSI-1436 has an effect on cellular sensitivity to insulin by modulating genes involved in insulin-stimulated glucose uptake signaling pathways. To further investigate this effect, a series of RT-qPCR analyses were performed. It was observed that there was a slight but not significant increase in GLUT2 insulin-dependent glucose uptake gene expression after MSI-1436 treatment in the investigated cells (Figure 2B). Furthermore, the expression of genes involved in insulin-stimulated glucose uptake, including AKT and PI3K, were examined. There were no significant changes observed in AKT expression between the groups of study (Figure 2D), but a decrease in PI3K expression was found after PA treatment compared to the control group of cells (*p* < 0.05), but further MSI-1436 treatment did not significantly alter the level of this gene in Eq_HPCs (Figure 2E). In addition, the expression of SIRT1 which plays a key role in the regulation of insulin secretion and sensitivity was examined [48]. This analysis showed a significant upregulation of SIRT1 in both treatments compared to the control (*p* < 0.0001). Moreover, MSI treatment significantly increased SIRT1 expression (Figure 2G) compared to PA-stressed cells (*p* < 0.0001). The results showed statistically significant increases in G6PD (Figure 2C) and mTOR (Figure 2F) transcript expression in PA-treated cells (*p* < 0.0001). However, MSI-1436 application resulted in a distinct subsequent downregulation of G6PD (*p* < 0.01) and mTOR (*p* < 0.001) compared to PA-treated cells.

Next, the protein expression of PTP1B and GLUT2 after PA and MSI-1436 treatment was investigated using SDS-PAGE and Western blot analysis (Figure 2H–K). The application of palmitate increased PTP1B protein expression (Figure 2K), reaching a statistically significant difference compared to control cells (*p* < 0.001). PTP1B works as a negative regulator for the insulin signaling pathways [49]. However, following MSI-1436 treatment of PA-stressed cells, a visible downregulation of PTP1B expression to comparable levels of the control cells (*p* < 0.001) was noted. Subsequently, the relative expression of the two GLUT2 isoforms, 34 kDa (Figure 2J) and 45 kDa (Figure 2I), in PA-stressed cells was significantly decreased (*p* < 0.001 and *p* < 0.0001, respectively) in relation to the control. Importantly, the application of MSI-1436 did not contribute to the restoration of GLUT2 basal levels (*p* < 0.0001 and *p* < 0.001, respectively) when compared to both control groups. Additionally, the potential effect of MSI-1436 treatment on glucose uptake was measured by intracellular glucose content using GC/MS analysis. The results showed that intracellular glucose concentration in PA-stressed cells and PA-treated cells with MSI-1436 revealed a significant increase compared to control Eq_HPCs (*p* < 0.01 and *p* < 0.01, respectively). Furthermore, a significant difference was observed between Eq_HPCs stressed and treated with MSI-1436, with *p* < 0.03 (Figure 2L,M). 

### 3.3. MSI-1436 Enhances Mitochondrial Dynamics in Eq_HPCs

**Figure 3:**
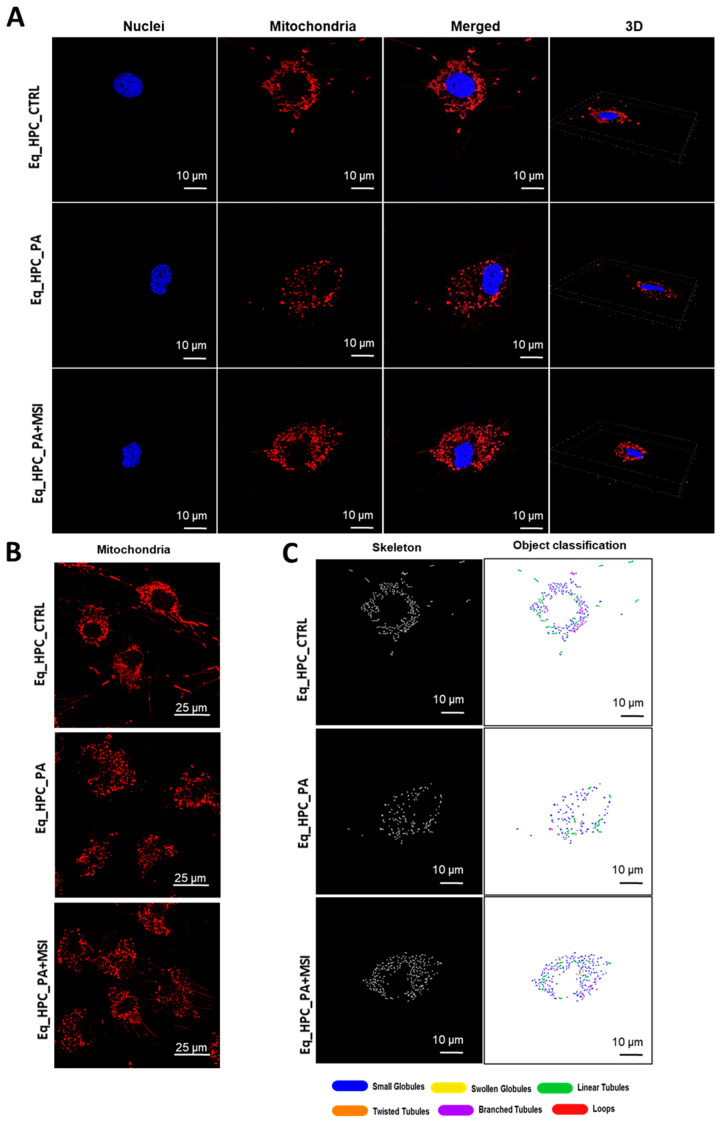

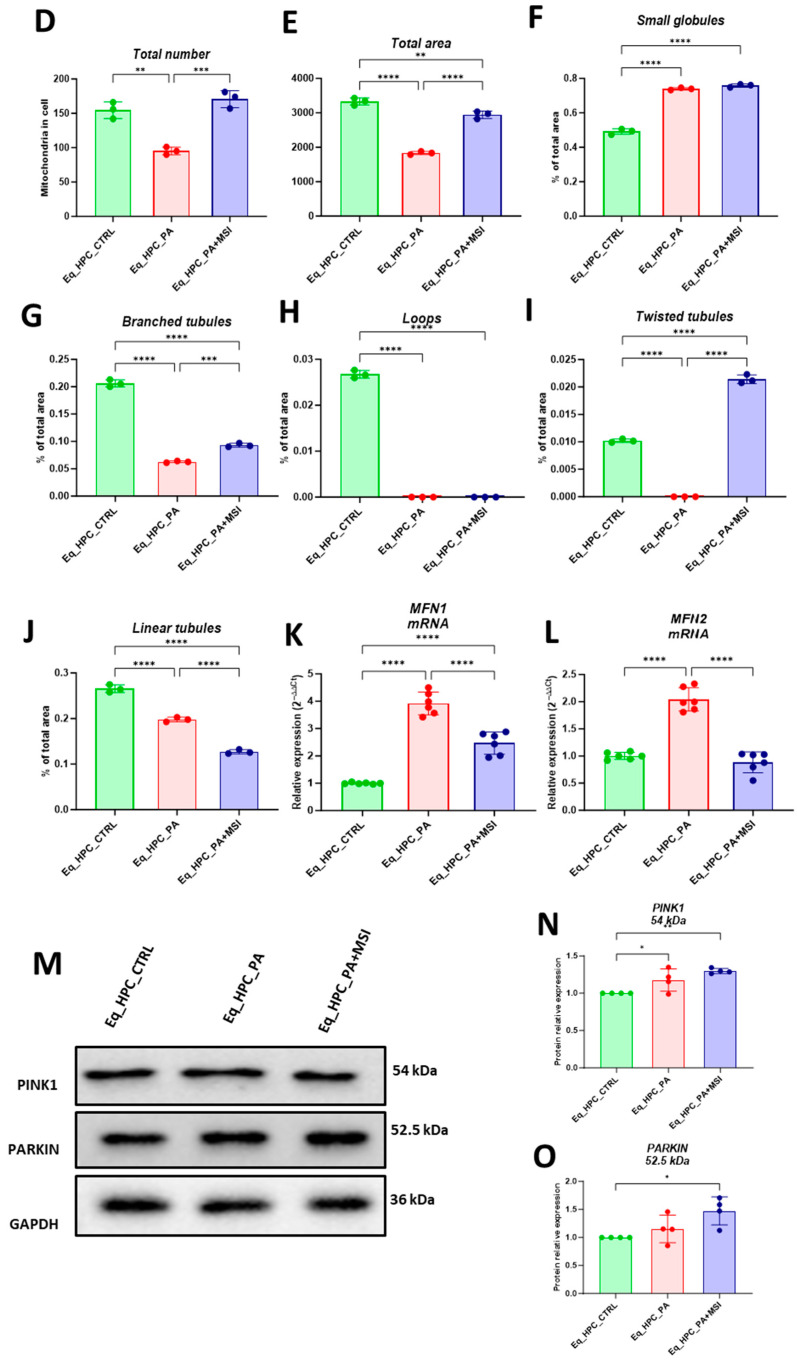
MSI-1436 effect on mitochondrial morphogenesis in Eq-HPCs. (**A**) The effect of PA- and MSI-treatment on cells stained with Mito Red. MitoGraph software was used to process the images. MitoGraph turns 3D bioimages into surfaces. (**B**) The node-and-edge structures (skeletons) from mitochondrial networks. (**C**) Images showing mitochondrial number and distribution in Eq-HPCs relative to cells after PA induction and MSI-1436 application. The bar graphs show morphological subtypes of mitochondria: (**D**) total number in the cell, (**E**) total area in the cell, (**F**) small globules, (**G**) branched tubules, (**H**) loops, (**I**) twisted tubules, and (**J**) linear tubules. (**K**) MFN1 and (**L**) MFN2 measured with RT-qPCR. The results from experiments are normalized to GAPDH mRNA levels and expressed as fold change over the non-treated group as the control. (**M**) Western blot analysis of PINK1 (54 kDa) and PARKIN (52.5 kDa) normalized to GAPDH (36 kDa). (**N**,**O**) The color code of the bars shows the corresponding changes in putative kinase 1 (PINK1) and Parkin in Eq_HPCs treated with PA and MSI-1436, which commonly interact in a mitophagy pathway initiated by loss of the mitochondrial membrane potential. Results are expressed as mean  ±  SD. Significant changes (* *p* < 0.05, ** *p* < 0.01, *** *p* < 0.001, and **** *p* < 0.0001) are marked with an asterisk.

Mitochondrial morphology transitions play a crucial role in regulating various metabolic processes and are closely linked to cellular stress and the disruption of homeostatic integrity. Therefore, this study investigated changes in mitochondrial architecture in Eq-HPCs after exposure to PA and treatment with MSI-1436 using fluorescence imaging. The mitochondria were visualized using the red fluorescent MitoTracker (Figure 3A–C); PA-exposed HPCs showed a significant decrease in the total number of mitochondria (Figure 3D) and a reduced density of mitochondria compared to healthy cells (*p* < 0.01 and *p* < 0.0001, respectively). However, pretreatment of HPCs with MSI-1436 prior to PA exposure prevented the deterioration of the mitochondrial network and maintained the proper density and total surface area of mitochondria, in contrast to the untreated cells (*p* < 0.001) (Figure 3E). 

Further analysis using the MicroP program categorized mitochondria into three types based on their morphological characteristics including small globular, branched, linear, twisted, and loop- or donut- shaped mitochondria. As shown in Figure 3F–J, PA exposure led to an increased number of small globular mitochondria and a decrease in loop-shaped mitochondria, which was not prevented by MSI-1436 pretreatment (*p* < 0.0001) (Figure 3F,H). Moreover, the data clearly indicated that PA induced significant fragmentation and fission of mitochondria, as evidenced by the lower proportions of mitochondria with linear, branched, and twisted tubules compared to the control cells (*p* < 0.0001) (Figure 3G,I,J). Interestingly, pretreatment of HPCs with MSI-1436 exhibited a higher number of branched tubular mitochondria (Figure 3G) and twisted tubular (Figure 3I) mitochondria compared to PA-stressed HPCs. Furthermore, MSI-1436 further increased the abundance of twisted tubules compared to healthy control cells (*p* < 0.0001), indicating its profound ability to stimulate mitochondrial biogenesis, fusion, and networking. To better understand the observed changes in the mitochondrial network, the gene expression of mitofusins 1 and 2 was determined. The results showed that PA exposure disrupted the gene expression of these key markers involved in mitochondrial fusion. PA-exposed cells exhibited overexpression of MFN1/2 compared to the control cells (*p* < 0.0001 and *p*>0.0001, respectively), which suggests an acute response to increased mitochondrial fission and the resulting abundance of fragmented globular mitochondria. Additionally, HPC pretreatment with MSI-1436 normalized the levels of MFN1/2 transcripts compared to both the control and PA groups (Figure 3K,L), indicating the regulation of mitochondrial dynamics. Increased mitochondrial damage triggers the activation of mechanisms for clearing defective organelles in order to reduce the load of dysfunctional mitochondria. In this study, the activation of the PINK/PARKIN mitochondrial degradation signaling pathway was further analyzed. PA-exposed cells showed unchanged levels of PARKIN protein and a slight increase in the expression of PINK1 protein compared to the control cells (Figure 3N,O), suggesting the initiation of early stages of mitophagy. Interestingly, pretreatment with MSI-1436 induced a more pronounced activation of both PINK1 and PARKIN proteins compared to both the control and PA-treated groups (*p* < 0.05).

## 4. Discussion

The significant rise in liver disease cases highlights the urgent need for effective therapeutic interventions. Integrating previous research findings, it is evident that PTP1B shows promise as a critical target for acute liver injury, non-alcoholic fatty liver disease (NAFLD), and hepatocellular carcinoma (HCC). It regulates key processes such as hepatocyte apoptosis [50], hepatic lipogenesis [51], insulin resistance [51,52,53], and ER stress response [54]. Therefore, PTP1B inhibitors like MSI-1436 have tremendous potential as therapeutic agents against liver disorders [55]. However, little is known about the molecular effects of the MSI-1436 inhibitor on hepatic progenitor cells (HPCs). Hence, this study aims to investigate the impact of the protein tyrosine phosphatase 1B inhibitor (MSI-1436) on glucose metabolism, mitochondrial dynamics, and biogenesis in equine hepatic progenitor cells (HPCs) stressed with palmitate.

The biological effects of saturated and unsaturated (monounsaturated and polyunsaturated) fatty acids are primarily determined by their chemical structure. This is especially relevant for the fatty acids commonly found in the diet, such as palmitic acid (PA) [26,56,57]. In addition, fatty acids (FAs) play a crucial role in the development of non-alcoholic steatohepatitis (NASH). Long-chain fatty acids (LCFAs) promote the accumulation of lipids, inflammation, and the production of reactive oxygen species in the liver. Specifically, palmitic acid (PA) is particularly lipotoxic to the liver [58,59]. In fact, PA has been linked to insulin resistance in cultured HepG2 cells [60,61]. Moreover, palmitic acid has been found to inhibit insulin sensitivity by promoting the overexpression of PTP1B, a phosphatase that hinders the insulin signaling network by dephosphorylating crucial proteins including the insulin receptor and its downstream substrate, insulin receptor substrate 1/2 (IRS-1/2) [62,63]. Impaired hepatic insulin signaling significantly contributes to the stimulation of gluconeogenesis while suppressing glycogen synthesis [64].

The regulation of hepatic glucose metabolism is a highly intricate process that involves multiple pathways, one of which is the phosphatidylinositol 3-kinase (PI3K)/protein kinase B (AKT) signaling pathway, which plays a crucial role [65]. In the liver, AKT activation is involved in several physiological processes [66]. AKT inhibits glycogen synthase (GS) kinase (GSK), leading to an increase in GS activity and subsequently stimulating glycogen production [67]. Additionally, AKT suppresses gluconeogenesis by inactivating forkhead box O1 (FoxO1), which reduces the expression of key gluconeogenic genes such as phosphoenolpyruvate carboxykinase (PEPCK), glucose-6-phosphatase (G-6-Pase), and fructose 1,6-bi-phosphatase (FBPase) [68,69,70]. 

AKT also regulates sterol regulatory element-binding protein 1 (SREBP1) to stimulate endogenous fatty acid synthesis [71]. Furthermore, AKT promotes glucose transporter 2 (GLUT2) to transport glucose from the periphery into the cells for aerobic metabolism or anaerobic degradation [72]. It is worth noting that hepatocytes mainly express the GLUT2 isoform for glucose transport, and the suppression of GLUT2 expression in hepatocytes has revealed a previously unknown glucose output pathway that may rely on a mechanism dependent on membrane traffic. However, the expression of GLUT2 remains crucial for regulating glucose-sensitive genes, and its deactivation in the liver has been observed to result in impaired insulin secretion triggered by glucose stimulation [73].

Considering that palmitic acid (PA) has been found to decrease glucose uptake while decreasing the expression of phosphoinositide 3-kinase (PI3K) and protein kinase B (AKT) mRNA in HepG2 cells [74], a targeted inhibitor of PTP1B could potentially regulate multiple genes involved in glucose metabolism through the IRS/Glut2/PI3K/AKT pathways.

Additionally, activation of the SIRT1 protein has been shown to upregulate genes associated with glucose metabolism, while knockdown of SIRT1 reduces glucose output in mice; for instance, in diabetic rats, the application of an antisense oligonucleotide to knockdown SIRT1 decreased basal gluconeogenesis and increased hepatic insulin responsiveness [75]. In this study, we have demonstrated that MSI-1436 significantly enhances glucose uptake in palmitate-treated HPCs leading to improved glucose metabolism through the G6DP and SIRT1/mTOR axis. It has been observed by others that the upregulation of glucose-6-phosphate dehydrogenase (G6PD) in the liver of obese and diabetic animals can exacerbate oxidative stress and impair tissue function, suggesting that abnormal G6PD expression in obesity may contribute to metabolic dysfunction by disrupting energy balance and redox homeostasis [76]. Furthermore, SIRT1, a nuclear-localized, NAD+-dependent protein deacetylase, regulates the acetylation of various non-histone proteins in hepatocytes [77,78,79]. For instance, SIRT1-mediated deacetylation of sterol regulatory element-binding protein 1c (SREBP1c) reduces the expression of lipogenic genes, such as acetyl-CoA carboxylases (ACC) and fatty acid synthase (FAS) [80,81].

Deacetylation of peroxisome proliferator-activated receptor-γ coactivator 1α (PGC-1α) enhances its activity, leading to increased transcription of carnitine palmitoyltransferase 1 (CPT1), which is necessary for mitochondrial fatty acid oxidation [82,83,84]. Additionally, deacetylation of liver kinaseB1 (LKB1) promotes its translocation to the cytoplasm and phosphorylation of AMP-activated protein kinase (AMPK), resulting in decreased lipogenesis through the mammalian target of rapamycin (mTOR)/ liver X receptor α (LXRα) signaling pathway and increased fatty acid oxidation through ACC phosphorylation [85]. Interestingly, our data showed that MSI-1436 treatment downregulates G6DP expression in palmitate-treated HPCs, thereby improving cellular redox homeostasis and glucose tolerance. Furthermore, MSI-1436 induces SIRT1 expression and decreases mTOR expression in HPCs. 

As further evidence of the effects of MSI-1436 on cellular metabolism, we observed increased glucose uptake with HPCs challenged with palmitate when treated with MSI-1436. This was demonstrated by 2-NBDG staining and glucose-GC/MS analysis. These findings suggest that MSI-1436 can effectively modulate cellular metabolism and energy homeostasis, making it a promising therapeutic agent for metabolic disorders. However, despite these effects, there was no increase in GLUT2 expression. Rémy Burcelin and his colleagues previously reported a similar observation in GLUT2-null hepatocytes, providing further evidence that supports the existence of a membrane-based glucose release pathway independent of Glut2 in hepatocytes and intestinal cells [86,87,88]. Therefore, the precise mechanisms underlying this phenomenon might be associated with a GLUT2-independent transport system, such as a membrane traffic-based pathway, leading to an increased glucose influx. 

Several in vitro studies have suggested that palmitate can induce oxidative stress and cause significant damage to mitochondrial DNA. This damage has been shown to be associated with mitochondrial dysfunction, apoptosis, and inhibition of insulin signaling [89,90,91]. Moreover, hepatic insulin resistance has been linked to hepatic fatty acid-induced mitochondrial dysfunction, which impairs mitochondrial function and energy metabolism [92,93,94,95,96]. Additionally, mitophagy, a catabolic process, can selectively remove damaged mitochondria, preserving mitochondrial function and reducing reactive oxygen species that cause mitochondrial dysfunction [97]. Therefore, promoting mitophagy may enhance fatty acid oxidation and reduce hepatic fatty acid accumulation, offering a potential therapeutic approach to protect against mitochondrial dysfunction [97]. In this study, we observed that the PTP1B inhibitor (MSI-1436) effectively maintained mitochondrial dynamics in HPCs that had been severely impaired by exposure to palmitate. Specifically, MSI-1436 increased both the total area and number of mitochondria and regulated the expression of mitochondrial fusion genes such as Pink1, Parkin, and Mnf1/2. Furthermore, the inhibitor promoted the development of morphological architecture of mitochondria, including branched and twisted tubules, which are characteristic of healthy mitochondrial function. Therefore, MSI-1436 plays a crucial role in maintaining mitochondrial quality control and preventing the accumulation of damaged or dysfunctional mitochondria through mitophagy activation, facilitating the clearance of damaged mitochondria. 

The obtained data are in good agreement with other in vivo studies demonstrating that administration of MSI-1436 to horses with equine metabolic syndrome (EMS) resulted in similar improvements in mitochondrial dynamics. Specifically, the protein tyrosine phosphatase 1B (PTP1B) inhibitor demonstrated a robust regulatory effect on the expression of MFN-2, PINK1, and PARKIN, confirming its efficacy in enhancing mitochondrial and overall liver metabolism under EMS conditions. Previous studies have shown that the deletion of PTP1B in mice also improves mitochondrial integrity by suppressing ER stress-mediated overexpression of Pink1 and Parkin, supporting the therapeutic potential of PTP1B inhibitors for the restoration or enhancement of mitochondrial biogenesis [98]. Another study showed induction of PINK1 and a reduction in PARKIN levels after MSI-1436 pretreatment in a human hepatocarcinoma cell line [5]. However, this contradiction with our results could be attributed to a cell type-dependent effect.

Collectively, these findings highlight the potential of PTP1B inhibitors in facilitating the restoration or enhancement of mitochondrial biogenesis, a fundamental process critical for maintaining cellular energy homeostasis and proper physiological function, as demonstrated by MSI-1436.

## 5. Conclusions

The present study characterizes, for the first time, the effects of MSI-1436 on the survival, glucose uptake, and mitochondrial morphogenesis of equine hepatic progenitor cells (HPCs). We have shown that the in vitro application of MSI-1436 promotes the viability of Eq_HPCs and provides protection against palmitate-induced apoptosis and insulin resistance. Our results strongly suggest that the enhanced glucose uptake observed in HPCs treated with MSI-1436 is mediated by the increased expression of the SIRT1 gene and the maximization of ATP production through the promotion of the mitochondrial morphotype. Clinical trials are necessary to confirm the utility of MSI-1436 in veterinary clinical medicine.

## Figures and Tables

**Table 1:** Primers used for gene expression analysis.

Gene	Primer	Sequence 5′–3′	Amplicon Length (bp)	Accession No.
* MFN1 *	F: R:	AAGTGGCATTTTTCGGCAGG TCCATATGAAGGGCATGGGC	217	XM_005601821.3
* MFN2 *	F: R:	AATGCCATGCTCTGGGACAA CATCAGCGTCCAGGCAAAAC	325	XM_023635773.1
* BAX *	F: R:	CGAGTGGCAGCTGAGATGTT AAGGAAGTCCAGTGTCCAGC	153	XM_023650076.1
* BCL2 *	F: R:	TTCTTTGAGTTCGGTGGGGT GGGCCGTACAGTTCCACAA	164	XM_001490436.4
* SIRT1 *	F: R:	ACCAACGGTTTTCATTCTTGTG ATTCGAGGATCTGTGCCAATCA	139	XM_023643979.1
* AKT1 *	F: R:	AAGGAGATCATGCAGCACC GCTCCATCGTGTCGTCTTGGT	180	XM_023628568.1
* PI3K *	F: R:	GACTTGCACTTGGGTGACATA TAAGTTCCCGGAAAGTCCCC	152	XM_023625590.1
* mTOR *	F: R:	GGGCAGCATTAGAGACGGTG ATGGTTGATTCGGTGTCGCA	221	XM_023635800.1
* G6PD *	F: R:	CAGAGCGAGCCCTTCTTCAA CAGGTAGTGGTCAATGCGGT	363	XM_023634095.1
* GAPDH *	F: R:	GATGCCCCAATGTTTGTGA AAGCAGGGATGATGTTCTGG	250	NM_001163856.1

Mfn1: mitofusin 1; Mfn 2: mitofusin 2; Bax: Bcl-2-associated X protein; Bcl-2: B-cell lymphoma 2; Sirt1: Sirtuin 1; Akt: serine/threonine 308 kinase; Pi3k: phosphoinositide 3-kinase; mTOR: mechanistic target of rapamycin; G6PD: glucose-6-phosphate dehydrogenase; GAPDH: glyceraldehyde-3-phosphate dehydrogenase.

**Table 2:** List of antibodies used in Western blot analysis.

Antibodies	Concentrations	CAT Numbers	Company
*GAPDH*	1:2500	ab9485	Abcam
*SIRT1*	1:5000	ARP32386	Aviva
*PINK 1*	1:250	orb331223	Biorbyt
*GLUT2*	1:500	orb10726	Biorbyt
*PTP1B*	1:1000	ARP45360	Aviva
*PARKIN*	1:250	NB100-91921	Novus

GADPH: glyceraldehyde-3-phosphate dehydrogenase; SIRT1: sirtuin 1; Pink1: PTEN-induced putative kinase 1; Glut2: glucose transporter 2; PTP1B: protein-tyrosine phosphatase 1B; Parkin: RBR E3 ubiquitin protein ligase (PARK2).

## Data Availability

The data that support the findings of this study are available from the corresponding author upon reasonable request.
